# Superficially invasive cervical squamous cell carcinoma metastatic to ovarian endometriotic cyst wall, a case report and brief review of the literature

**DOI:** 10.1186/s13048-018-0417-9

**Published:** 2018-05-30

**Authors:** Minfen Zhang, Elena Lucas, Hanzhen Xiong, Shaoyan Liu, Kyle Molberg, Qingping Jiang, Wenxin Zheng

**Affiliations:** 1 Department of Pathology, Third Affiliated Hospital, Guangzhou Medical University, Guangzhou, 510150 People’s Republic of China; 2 Key Laboratory of Major Obstetric Diseases of Guangdong Province, The Third Affiliated Hospital, Guangzhou Medical University, Guangzhou, 510150 China; 3 Department of Pathology, University of Texas Southwestern Medical Center, Dallas, TX 75390 USA; 4 Department of Obstetrics and Gynecology, University of Texas Southwestern Medical Center, Dallas, TX 75390 USA

**Keywords:** Uterine cervix, Superficial invasive squamous cell carcinoma, Ovarian metastasis, Ovary, Endometriosis

## Abstract

**Background:**

Although cases of cervical squamous cell carcinoma metastatic to the ovary have been previously documented, we report the first case of superficially invasive squamous cell carcinoma metastatic to the ovary.

**Case presentation:**

A 45-year-old woman with a two-year history of ovarian endometriosis confirmed by ultrasound underwent oophorectomy. On microscopic examination, a focus of malignant stratified epithelium, initially interpreted as transitional cell carcinoma, was identified within the endometriotic cyst wall. Examination of the hysterectomy specimen revealed superficially invasive squamous carcinoma of the cervix. In addition, two triploid, CD45-negative cells were detected during the analysis of the peripheral blood for circulating tumor cells (CTC). High-risk HPV was detected on the sections of endometriosis containing cancerous area by using hybrid capture 2 assay, supporting the diagnosis of metastatic squamous cell carcinoma originating from the uterine cervix.

**Conclusion:**

This is the first report of superficially invasive squamous cell carcinoma metastatic to the ovary. Such finding could be misdiagnosed as primary ovarian transitional cell carcinoma, squamous cell carcinoma originating from metaplastic epithelium within endometriosis, or squamous cell carcinoma arising in a teratoma.

## Background

Ovarian metastases from cervical squamous cell carcinoma (SCCA) are rare. They account for less than 1% of metastatic tumors in the ovary and typically occur in advanced stage cervical carcinoma. Only rare cases of ovarian metastasis from superficially invasive SCCA have been documented in the English literature [1–5]. To our knowledge, no cases of SCCA metastases to the ovary involving structures other than native ovarian tissue have been previously documented. We report a case of an incidental metastatic cervical superficial squamous cell carcinoma to the ovarian endometriosis.

## Case presentation

A 45-year-old woman presented for a routine physical examination. Her pelvic ultrasound revealed a 4.2 cm left ovarian cyst. Initially, the lesion was managed conservatively with observation. Over the next 2 years, the patient remained free of symptoms; however, her ovarian cyst doubled in size measuring 8.1 cm by ultrasound. A laparoscopic left oophorectomy was ultimately performed.

### Pathologic findings

**Fig. 1:**
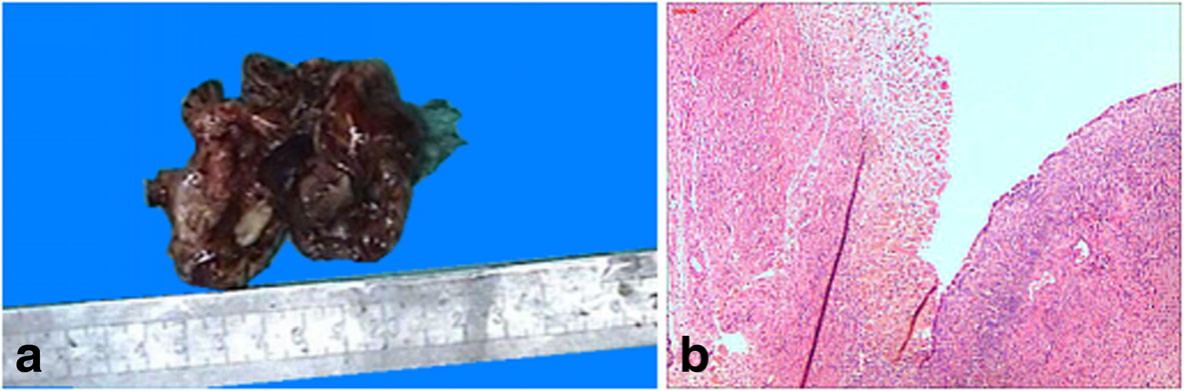
Gross and microscopic findings in the right ovarian cyst during intraoperative consultation. **a** Gross examination showed fragments of the hemorrhagic cyst wall. **b** Microscopic section demonstrated endometriosis (H&E, × 100)

**Fig. 2:**
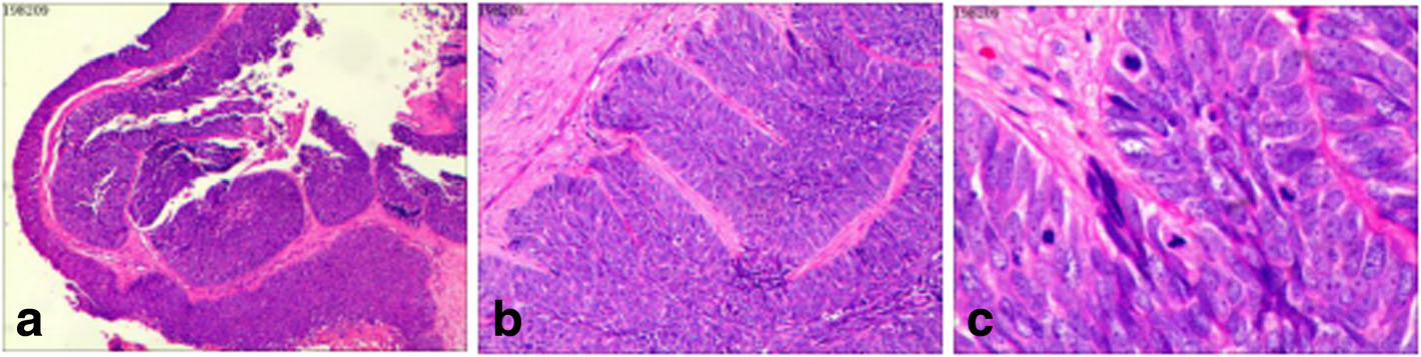
Carcinoma within endometriotic cyst wall. **a** Malignant epithelium lining the cyst wall and forming nests in the subepithelial stroma (H&E, × 40); **b, c** Malignant cells demonstrate large, hyperchromatic nuclei with irregular nuclear contours, prominent nucleoli, scant cytoplasm, and increased mitoses (H&E, B:× 100; C × 200)

Intraoperative pathologic evaluation revealed dark red cyst wall fragments, 7 cm in aggregate, and an unremarkable fallopian tube (Fig. 1a). The frozen section diagnosis was ovarian endometriosis (Fig. 1b), confirmed by evaluation of permanent sections. Among multiple additional permanent sections, several sections demonstrated atypical stratified epithelium in the subepithelial stroma within the cystic wall. The atypical cells had large, hyperchromatic nuclei, irregular nuclear contours, prominent nucleoli, scant cytoplasm, and numerous mitoses, consistent with malignant cells (Fig. 2a-c). The total size of malignant epithelium was approximately 15 mm. The remainder of the specimen was entirely submitted for microscopic examination and demonstrated ovarian tissue with endometriosis and an unremarkable fallopian tube. No evidence of teratoma was identified.

**Fig. 3:**
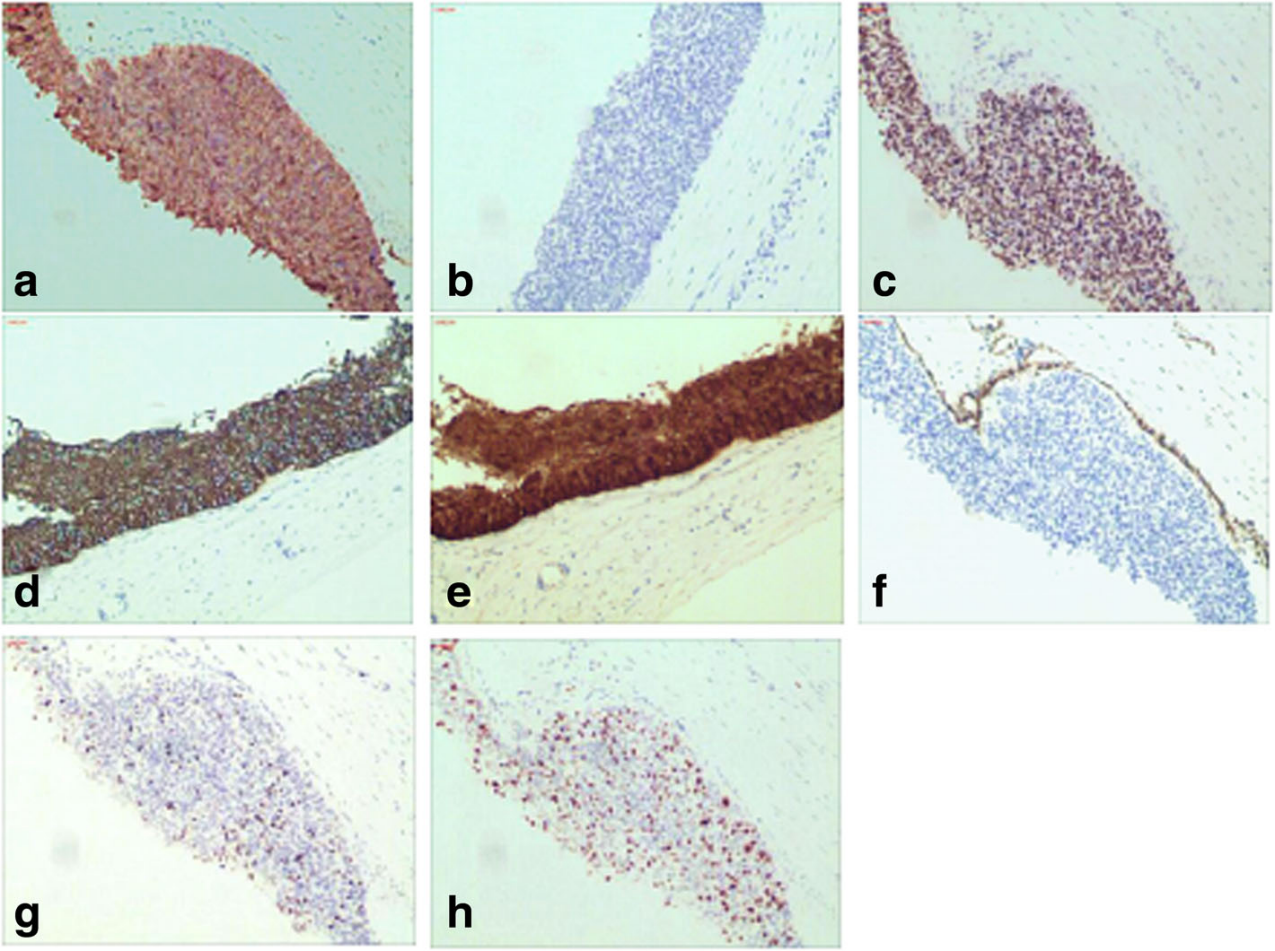
Immunohistochemical stains in the malignant epithelium. **a** Positive CK7; **b** Negative CK20; **c** Positive p63; **d** Positive CK5/6; **e** Positive p16; **f** Negative WT1; **g** p53 demonstrates wild-type staining pattern; **h** Ki67 proliferative index is approximately 50% (IHC, × 100)

By immunohistochemistry (IHC), the malignant cells were diffusely positive for CK7, CK5/6, p63, and p16, and negative for CK20, WT1, GATA3, ER, and PR. p53 demonstrated wild-type staining pattern. Ki67 proliferation index was approximately 50%. (Fig. 3a-h).

Based on the morphologic features and immunohistochemical stain findings, the case was diagnosed as ovarian transitional cell carcinoma-like high-grade serous carcinoma. A differential diagnosis of metastatic urothelial carcinoma of the urinary tract was entertained; however, no lesions were identified in the urinary tract by ultrasound or computerized tomography (CT) scan. To rule out squamous cell carcinoma arising from teratoma, the entire specimen was examined. No evidence of teratoma was identified. The patient sought external pathology consultations from two large regional medical centers, both of which agreed with the original diagnosis.

**Fig. 4:**
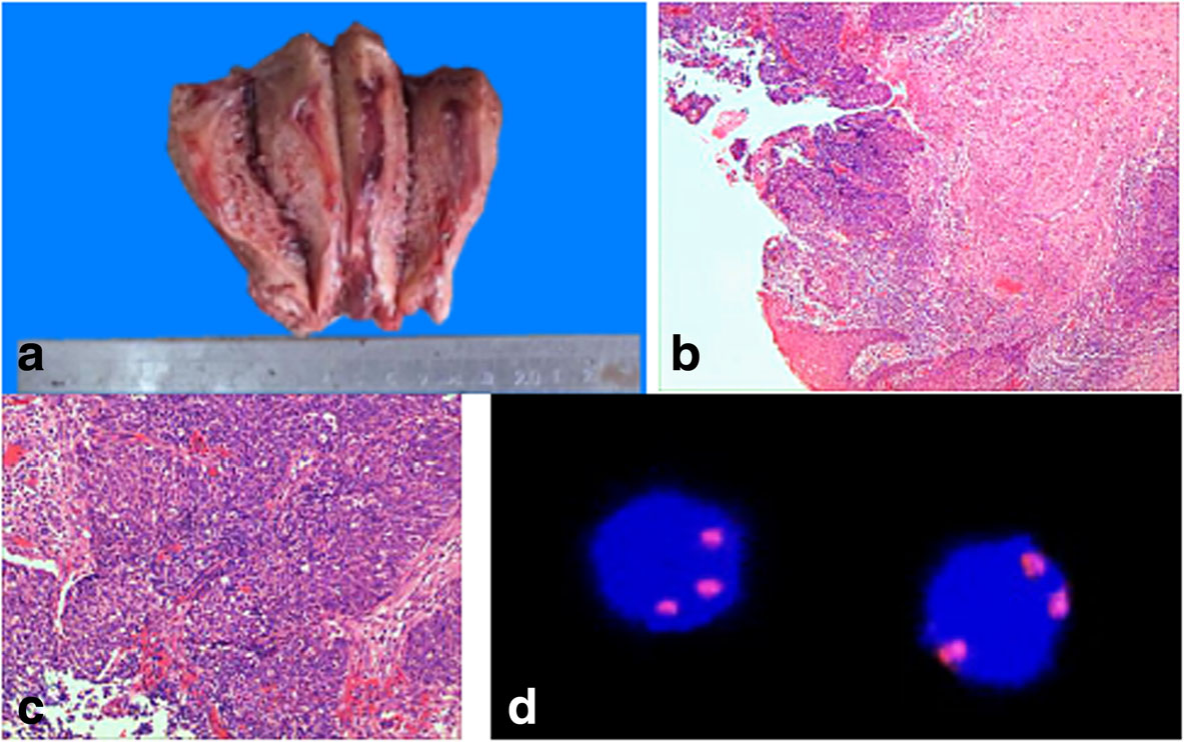
Uterine cervix with HSIL with focal superficially invasive squamous carcinoma. **a** Gross examination of the uterus demonstrated no abnormalities; **b, c** Sections of the cervix demonstrated HSIL with focal superficially invasive squamous carcinoma (H&E, × 100). **d** Analysis of the peripheral blood for circulating tumor cells detected two triploid CD45-negative malignant cells by FISH (× 400)

Total hysterectomy and right salpingo-oophorectomy with pelvic lymph node dissection and omentectomy were performed. Grossly, the uterus, cervix, right ovary and fallopian tube were unremarkable (Fig. 4a). Microscopically, the right ovary, right fallopian tube, and other specimens revealed no evidence of malignancy. Interestingly, representative sections of the cervix revealed high-grade squamous intraepithelial lesion (HSIL). Eventually the cervix was entirely submitted for microscopic examination. Additional cervical sections demonstrated HSIL with focal superficially invasive squamous cell carcinoma. The depth of invasion was 4.0 mm and a horizontal extent was 6.0 mm involving only one of total 12 sections of the cervix. No lymphovascular invasion was identified. Examination of the right ovary revealed no evidence of teratoma. The FIGO tumor stage was IA2 (Fig. 4b-c). Prior to hysterectomy, testing for high-risk HPV was performed on the cervical cytology specimen by hybrid capture2 (HC2) method (Qiagen Inc., USA), and was positive (935 RLU; reference range: < 1.00RLU).

The original diagnosis of transitional cell carcinoma-like high-grade serous carcinoma was questioned. To correlate the findings in the ovary and cervix, DNA extraction was performed from the ovarian sections with carcinoma. The DNA extraction and purification was performed according to the manufacturer’s instructions using GenElute™ FFPE DNA Purification Kit (MilliporeSigma, Burlington, USA). Testing for high-risk HPV was performed by HC2 method was positive (141.3 RLU; reference range: < 1.00 RLU).

To further support the diagnosis of metastatic carcinoma, analysis of the patient’s peripheral blood for circulating tumor cells (CTC) was performed. Several malignant, triploid, CD45-negative epithelial cells were identified, suggestive of the presence of carcinoma cells in the peripheral blood (Fig. 4d).

The final diagnosis was metastatic cervical squamous cell carcinoma involving ovarian endometriosis.

## Discussion

Ovarian metastases from cervical carcinoma occur in 5.3–8.2% cases of endocervical adenocarcinoma and 0.4–1.3% of squamous cell carcinoma [2, 6, 7]. The possible routes of cervical cancer spread to the ovary include direct extension, lymphatic and hematogenous spread, and trans-tubal migration. A few factors increase the risk for ovarian metastases. These include two independent factors, cancer type (adenocarcinoma greater than squamous cell carcinoma) and involvement of the uterine corpus. Additional factors increasing metastatic potential are vaginal involvement, lymphovascular invasion and lymph node metastases [6, 8].

Ovarian involvement is directly related to the stage of cervical carcinoma. It ranges from 0.22% for stage IB squamous cell carcinoma to 9.8% for stage IIB adenocarcinoma [2, 9, 10]. Stage IA carcinomas metastasize to the ovaries rarely, with only a few reports in the literature [11]. Young et al. reported a case of squamous cell carcinoma in situ with right ovarian surface involvement [12]. But, the majority of reported cases describe advanced stage carcinomas involving the ovary after the diagnosis of cervical carcinoma was previously established in cervical LEEP or cold knife cone excision specimens. In our patient, the ovarian metastasis was an incidental finding. This case illustrates the importance of cervical cancer screening regardless of the presence of symptoms.

An additional, interesting finding was the involvement of the endometriotic cyst wall by carcinoma with sparing of the normal ovarian parenchyma. To our knowledge, this is the first documented case describing this unusual pattern of metastasis. The metastatic focus was very small and could be potentially overlooked. It was not visible on gross examination due to its small size, obscuring hemorrhage and discoloration due to endometriosis. In our case, the frozen section did not contain the metastasis. In addition, due to the rarity of squamous cell carcinoma metastases to the ovary and the overlapping immunostaining pattern (strong positivity for p16 and CK7), the tumor was initially misclassified as primary ovarian transitional cell-like high-grade serous carcinoma [13–17]. The diagnosis of metastatic squamous cell carcinoma was eventually established with the aid of the high-risk HPV test, which was positive in both the cervical and ovarian carcinoma.

## Conclusion

This is the first report of microinvasive cervical squamous cell carcinoma with a metastasis involving exclusively an ovarian endometriotic cyst wall. In the absence of a prior abnormal cervical screening history and any visible cervical lesions, such a case may be readily misdiagnosed as another type of carcinoma. In our case, a definitive diagnosis was successfully established by careful examination of the entire uterine cervix and comparing the HPV status of the primary and metastatic tumors. This case highlights the morphological similarity between SCCA and other types of primary ovarian cancer. A high index of suspicion is necessary to avoid a misdiagnosis of transitional cell carcinoma, high-grade serous carcinoma, poorly differentiated carcinoma, or squamous cell carcinoma arising from teratoma. Testing for high-risk HPV is helpful in establishing the correct diagnosis.

## Abbreviations

CT: Computed tomography
CTC: Circulating tumor cells
FFPE: Formalin fixed paraffin embedded
FIGO: International Federation of Gynecology and Obstetrics
H&E: Hematoxylin-eosin
HC2: Hybrid Capture 2
HPV: Human papillomavirus
HSIL: High-grade squamous intraepithelial lesion
IHC: Immunohistochemistry
LEEP: Loop electrosurgical excisional procedure
SCC: Squamous cell carcinoma
US: Ultrasound
